# Increased C-Reactive Protein Concentrations During Menstruation May Be Important for the Pathophysiology of Endometriosis and Possibly for Adhesion Formation—A Systematic Review

**DOI:** 10.3390/jcm15051711

**Published:** 2026-02-24

**Authors:** Maria Mercedes Binda, Maya Sophie de Wilde, Rudy Leon De Wilde, Philippe Robert Koninckx

**Affiliations:** 1 Department Obstetrics and Gynaecology, Katholieke University Leuven, 3000 Leuven, Belgium; mercedes.binda@gmail.com; 2 Rommel Consulting Partners SRL, 1370 Jodoigne, Belgium; 3 Department Obstetrics and Gynaecology, University Hospital for Gynaecology, Pius Hospital, University Medicine Oldenburg, Carl von Ossietzky University, 26121 Oldenburg, Germany; sophie.dewilde@pius-hospital.de (M.S.d.W.); rudy-leon.dewilde@pius-hospital.de (R.L.D.W.); 4 Department Obstetrics and Gynaecology, University of Oxford, Oxford OX1 2JD, UK; 5 Department Obstetrics and Gynaecology, University Cattolica del Sacro Cuore, 00168 Rome, Italy; 6 Departments of Obstetrics and Gynecology, Moscow State University, 119991 Moscow, Russia

**Keywords:** dysmenorrhoea, CRP, postoperative adhesions, acute inflammation, retrograde menstruation, endometriosis

## Abstract

**Objectives**: The peritoneal cavity is a cavity outside the bloodstream, with a specific hormonal, immunological and microbiological micro-environment distinct from plasma. The mesothelial cells lining the peritoneal cavity react within seconds to minor trauma, such as blood, with retraction, acute inflammation and later inflammation. This mesothelial cell retraction exposes the basal membrane, facilitating the implantation of tumour cells. Acute inflammation enhances adhesion formation after surgery and causes pain. The aim of the review was to check the hypothesis that retrograde menstruation, occurring in most women, is sufficient to cause some peritoneal irritation. **Design**: A systematic review of menstrual C-reactive protein (CRP) concentrations, a non-specific marker of peritoneal inflammation (PROSPERO ID 536306). **Results**: All articles (n = 8) showed a variable increase in CRP concentrations during the menstrual and early follicular phase of 80 ± 36%. **Conclusions**: CRP concentrations are slightly increased during menstruation and the early follicular phase. This increase is likely due to retrograde menstruation, causing mesothelial cell retraction and acute pelvic inflammation. It seems logical that mesothelial cell retraction facilitates endometrial cell implantation and accounts for the anatomical distribution of endometriosis lesions. Acute pelvic inflammation may enhance postoperative adhesion formation.

## 1. Introduction

Retrograde menstruation occurs in most women, as recently reviewed [1]. In the peritoneal fluid of women without and with endometriosis, endometrial cells were found in 54% and 75%, respectively [2], and peritoneal fluid is blood-stained in over 90% [3]. Retrograde menstruation, which contains viable endometrial cells with DNA similar to that of the endometrium [3], has been considered to support the implantation theory of endometriosis [4]. Also, dysmenorrhoea was suggested to be caused by retrograde menstruation and peritoneal inflammation [5]. Unfortunately, the volume of retrograde menstruation in individual women is unknown since it is difficult to measure, and it remains debated whether heavy menstrual bleeding, likely associated with more retrograde menstruation, increases the risk of endometriosis.

C-reactive protein (CRP) is an acute-phase protein secreted by the liver following interleukin-6 secretion by macrophages and T cells, signalling inflammation or tissue damage. It is a non-specific inflammation biomarker in many diseases, including cardiovascular, respiratory, gastrointestinal, musculoskeletal, neurological, autoimmune, and infectious diseases. Following gynaecologic or abdominal surgery, CRP concentrations increase significantly in proportion to the severity of the procedure and serve as a biomarker for postoperative complications associated with peritoneal inflammation [6,7]. However, with the introduction of more sensitive CRP assays, CRP has become investigated as a biomarker of systemic inflammation reflecting cardiovascular disease [8], endometriosis [9] and even follicular development [10]. However, it is unclear whether a significant increase of less than two-fold, and an increase to more than 50 mg/mL reflect similar clinically relevant mechanisms.

The peritoneal cavity is a cavity outside the bloodstream, similar to the mouth, and is lined by large, flat mesothelial cells. The peritoneal cavity is a specific microenvironment, with the lining mesothelial cells actively regulating the transport of liquids, electrolytes, proteins, gases, and cells between plasma and peritoneal fluid [11]. In women without ovarian activity, transudation of peritoneal fluid is minimal. In ovulatory women, the peritoneal fluid volume increases exponentially during the follicular phase and remains high during the luteal phase by exudation from the growing follicle or the corpus luteum. This ovarian exudation explains that estrogen and progesterone concentrations are much higher in peritoneal fluid than in plasma, with an acute increase after ovulation due to the release of follicular content into the peritoneal cavity. The peritoneal cavity is thus a specific microenvironment with higher estrogen and progesterone concentrations than in plasma [12]. Also, the immunology is different [13], with distinct microbiota from the genital tract and the bowel, and a low-grade inflammation in women with endometriosis [14,15,16,17,18,19,20].

**Figure 1:**
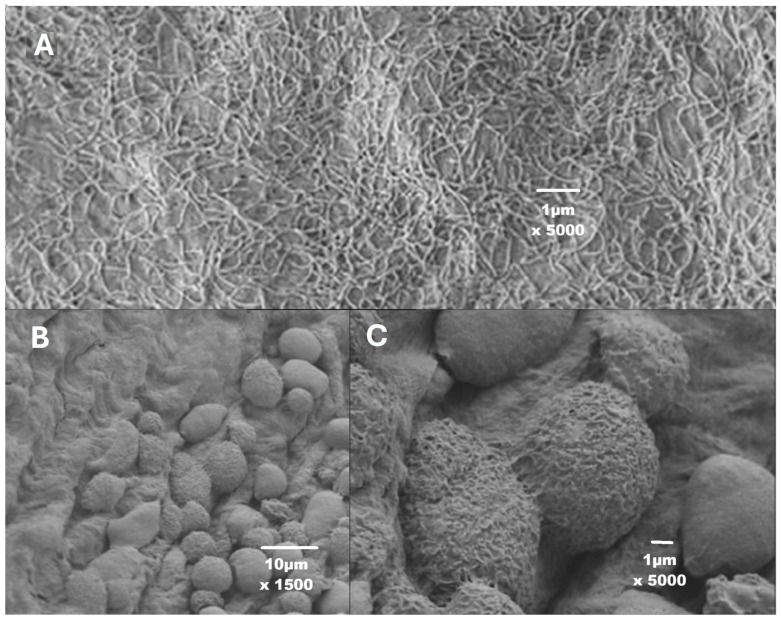
The mesothelial cells lining the peritoneal cavity are large (50 to 60 µm in diameter), flat cells with microvilli ((**A**) magnification 5000). These cells react within seconds to minor trauma by retracting, resulting in cell bulging ((**B**), magnification 1500), exposure of the basal membrane ((**C**), magnification 5000), and acute inflammation. Reproduced with permission from [22].

The mesothelial cells lining the peritoneal cavity respond within seconds (Figure 1) to minor trauma by retracting and bulging of cells, thereby exposing the basal membrane between cells [21,22,23]. The retraction of mesothelial cells facilitates the implantation of cells, such as tumour cells [24], and is associated with acute inflammation [25], the body’s initial response to harmful stimuli, characterised by increased transudation of many substances from plasma and diapedesis of white blood cells [26]. Acute inflammation triggers the liver to secrete CRP, which increases after 6 h. Even a minor trauma in the upper abdomen triggers acute inflammation with the release of substances into the peritoneal fluid, which enhances adhesion formation between surgical lesions in the lower abdomen [11]. A surgical lesion starts a cascade of events to repair the mesothelial defect. If mesothelial repair has not been completed by day 3, fibroblasts begin to grow and form adhesions, using any remaining fibrin as a scaffold [11]. Adhesion formation thus results from slower repair due to infection, necrotic tissue, or suture material. However, quantitatively equally important for adhesion formation is the severity of the acute inflammation of the entire peritoneal cavity, which is always associated with surgery since caused by minor trauma such as the insufflation pressure [27], duration of mesothelial hypoxia by CO_2_ pneumoperitoneum, oxidative stress of open surgery, desiccation, irrigation liquids such as saline [28], surgical manipulation of tissues, or blood [29]. Blood is highly irritant, causing mesothelial cell retraction, pain, and adhesion formation, and, in a mouse model, as little as 0.125 mL of blood or plasma can strongly increase adhesion formation [29].

Unfortunately, the importance of enhanced adhesion formation between surgical lesions, by mesothelial cell retraction and factors in peritoneal fluid, has only been demonstrated in mice without demonstrating a relationship with CRP concentrations. However, the observations are strikingly similar to the recommendations of microsurgery, emphasising gentle tissue handling, continuous irrigation, bloodless surgery, and dexamethasone at the end of surgery [30]. Remarkably, in mice, dexamethasone is only effective in preventing adhesions when the trauma to the mesothelial cell has been minimised by peritoneal conditioning.

Although a relationship between CRP concentrations and postoperative adhesions has not been demonstrated in humans, our hypothesis was that retrograde menstruation, occurring in most women [1], could cause mesothelial cell retraction and acute inflammation [21,22,23] since minimal amounts of blood are highly irritative. Moreover, retrograde menstruation also carries microbiota from the upper genital tract, potentially contributing to the inflammatory response. Since CRP secretion is a marker for acute inflammation and inflammation, we reviewed CRP concentrations during menstruation and the early follicular phase.

## 2. Materials and Methods

### 2.1. The Systematic Review

The aim of the study was to review the menstrual CRP concentrations as an indirect marker of retrograde menstruation. Therefore, the PubMed database was searched for studies on menstrual and early follicular CRP concentrations or peritoneal inflammation. This systematic review was registered in PROSPERO (ID 536306), and PRISMA guidelines for a systematic review were followed [31]. Articles in English, Spanish or Italian published before 24 November 2025, were considered. The first search (menstruation OR menses) AND inflammation AND (C-reactive protein OR CRP) found 50 articles. The second search (menstruation OR menses) AND (postoperative adhesions) found 101 articles. Since only eight articles were found by searching manually (MMB and PRK) the titles and abstracts for data on menstrual and early follicular CRP concentrations, an additional search was performed with (menstruation OR menses [Title/Abstract]) AND (inflammation OR C-reactive protein OR CRP [Title/Abstract]) AND human, but no additional data were found in these 988 articles.

Only 8 articles were found reporting menstrual CRP concentrations (Supplementary Figure S1). The aims of the articles were heterogeneous, and the studies were not designed to investigate retrograde menstruation or menstrual CRP concentrations, rendering a quality assessment of each study obsolete. Therefore, although complete, this review is not a systematic review, defined as a structured research method that identifies, assesses, and synthesises all available high-quality evidence on a specific, clearly defined research question to provide an unbiased, comprehensive answer, minimising bias through transparent, repeatable protocols and methods as used in evidence-based medicine and meta-analyses. The data were analysed as a comparative pooled analysis [32] to combine the results of a small number of heterogeneous studies. Since a comparative pooled analysis is not listed as a type of article (MDPI guidelines), the title mentions ‘systematic review’ to emphasise that all articles describing menstrual changes in CRP concentrations and published before 24 November 2025, were included without inclusion or exclusion criteria, except for interfering diseases or surgery.

### 2.2. C-Reactive Protein Assays

The concentrations of the annular CRP of 120,000 dalton are assayed with an immunoassay with within and between assay variances of 4 to 10% and within individual variances of 1.6% [33]. Traditional assays detect CRP concentrations in the range of 10 to 1000 mg/L. High-sensitivity CRP (hs-CRP) assays detect CRP in the range of 0.5 to 10 mg/L but are more expensive and take longer. Results will moreover vary with the test and antibodies used.

CRP concentrations were measured in blood. Although results in serum and in plasma can vary slightly between assays and concentrations this has not been taken into account since it does not affect the result of percentage increase. CRP concentrations were measured with hs-CRP assays in all manuscripts except in 3 [34,35,36]). To compensate for variability across assays and reporting, the menstrual changes in CRP concentrations across studies were compared as percentage increases rather than absolute values. Most articles reported medians and interquartile ranges; two articles (Table 1) reported the means and SDs. Also, the logarithmic distribution of plasma hormone concentrations [37] was never considered.

### 2.3. Statistics

The retrieved data from the eight selected articles are listed in Table 1. The menstrual increase of CRP concentrations was calculated by dividing the mean or median menstrual or early follicular concentrations by the respective late follicular or luteal phase concentrations. One article with serial CRP measurements during the cycle only listed the percentage change compared to the mean CRP concentration [38]. In one article, data had to be extracted from the figure [39].

The mean and standard deviations or standard error of the mean were calculated using SAS software [40]. The variability in sampling does not affect the conclusion that CRP concentrations increase during menstruation. However, to evaluate the variability of this increase across cycles within individual women or in the mean increase between women, specifically designed studies will be necessary.

**Table 1:** Menstrual or early follicular (EF) and late follicular (LF), and luteal (L) CRP concentrations (mg/L) and the reported *p*-values. The reported data were highly variable. Data were reported as mean and standard deviation (SD), median and interquartile range (IQR) or minimum and maximum values. N reflects the number of samples analysed. However these samples were taken in one menstrual cycle of different women, or in several cycles of the same women. This is indicated as N (p), where N reflects the number of samples analysed and p the number of women if more than 1 sample/patient was taken. Ref. [39] evaluated 1 sample in 9 and 2 samples in 250 women. The percentage increase was calculated for each report separately.

	Menstrual or EF			Late Follicular or Luteal			*p* Value	% Increase (Mean ± SD)
	N (p)	Mean (Median)	SD (SQR) [Min–Max]		Mean (Median)	SD (SQR) [Min–Max]		
Talebpour et al. 2023 [41]	111 (37)	(0.4)	[0.0–4.0]	L	(0.2)	[0.0–2.0]	-	100
Yama et al. 2020 [34]	21	(0.24)	(0.1–0.4)	L	(0.18)	(0.1–0.6)	NS	33
Chaireti et al. 2015 [42]	102	1.65	3	L	0.89	1	0.025	85
Gursoy et al. 2015 [35]	27	(1.8)	[0.3–7.7]	LF	(0.7)	[0.1–8.3]	<0.001	157
Gaskins et al. 2012 [39]	509	(0.75)	(0.4–1.7)	LF	(0.45)	(0.3–1.2)	0.001	66
Wander et al. 2008 [36]	72 (8)	2.8	-	LF	1.7	-	0.01	64
Puder et al. 2006 [43]	15	1.4	1.3	LF	0.8	0.74	0.007	75
Blum et al. 2005 [38]	15	130%	50%	LF	78%	30%	0.00001	67
NS: not significant								80 ± 36

## 3. Results

### 3.1. Menstruation and CRP

**Figure 2:**
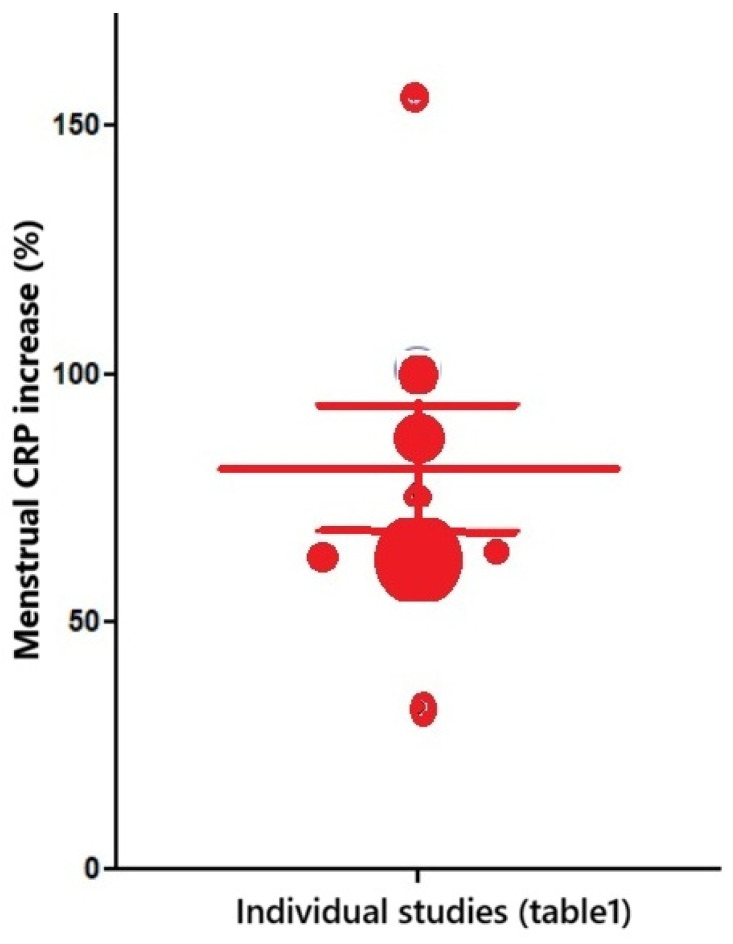
Menstrual or early follicular (EF) increase in CRP concentrations in comparison with late follicular (LF) and luteal (L) concentrations. The size of the bubbles is proportional to the number of subjects or the number of cycles investigated. The mean and standard error of the mean are indicated, but do not reflect the variable number of women included.

During menstruation and the early follicular phase, plasma CRP concentrations consistently increase and almost double (Table 1 and Figure 2). This observation received little attention, as menstrual inflammation was never the primary aim of these publications. Blum et al. [38] focused on the relationship of CRP with obesity and insulin resistance. Puder et al. [43] described the correlation between menstrual psychological and physical symptoms and CRP concentrations. Wander et al. [36] focused on changes in a group of hormones and CRP concentrations during the entire menstrual cycle. Gaskins et al. [39] is the largest series showing median and interquartile ranges of CRP and other hormones during the menstrual cycle. The increased CRP concentrations in the early follicular phase were subsequently confirmed by Gursoy et al. [35], Chaireti et al. [42], Talebpour et al. [41] and Yama et al. [34], the latter focusing on premenstrual syndrome (PMS).

### 3.2. Menstruation, Inflammation and Adhesion Formation

No data were found describing the volume of retrograde menstruation. Therefore, there are no data demonstrating that the volume of retrograde menstruation is related to the menstrual increase in CRP concentrations.

No data were found, indicating that the menstrual increase in CRP concentrations might be associated with postoperative adhesion formation as suggested by indirect evidence [44]. Hellebrekers et al. demonstrated in patients undergoing abdominal myomectomy that, at second-look laparoscopy, CRP concentrations correlated with the incidence of adhesions and the total amount of adhesions in the pelvis and to the uterus [44].

### 3.3. Logarithmic Distribution of CRP Concentrations

The logarithmic distribution of CRP concentrations can be concluded from the medians and quartile ranges and from means minus 3 SDs being below zero (Table 1).

## 4. Discussion

The menstrual increase in CRP concentrations is consistently reported across publications (Figure 1): CRP concentrations almost double, except in one study, which reported only a 33% increase [34]. Remarkably, this clear increase in menstrual CRP concentrations has received little attention, probably explained by these publications focusing on menstrual symptoms such as psychology, dysmenorrhea, cardiovascular accidents, premenstrual syndrome, polycystic ovarian syndrome (PCOS) and metabolic syndrome. Moreover, for surgeons, a 2-fold increase in CRP concentrations, as observed during follicular development [10] and in cardiovascular disease [8], seemed less important than the severe increases during surgical complications.

The increased CRP concentrations during menstruation and in the early luteal phase are likely caused by retrograde menstruation, since it is unlikely that blood from retrograde menstruation would not cause mesothelial cell retraction and acute inflammation in the pelvis (Figure 1), given the sensitivity of mesothelial cells to minor amounts of blood, as demonstrated in mice [29]. Since the increase in CRP concentrations persists during the early follicular phase, the uterine inflammation during menstruation [45,46] seems a less likely explanation. Unfortunately, no data are available to confirm the absence of a menstrual increase in CRP concentrations in women with occluded tubes. The hypothesis that retrograde menstruation triggers mesothelial cell retraction, acute inflammation and elevated CRP concentrations is clinically important. First, the severity of the inflammatory reaction will vary with the amount of retrograde menstruation, and the consequences will be individually variable. Second, this hypothesis sheds new light on our understanding of the pathophysiology of endometriosis. Mesothelial cell retraction, exposing the basal membrane, will facilitate the implantation of endometrial cells or fragments, as demonstrated by the increased tumour cell implantation [24]. This facilitated implantation of endometrial cells is consistent with clinical observations, such as the widely held belief that women with endometriosis have heavier menstrual bleedings, with a higher probability of having more retrograde menstruation and more pelvic pain. The hypothesis of retrograde menstruation, mesothelial cell retraction facilitating implantation, is also consistent with the anatomical localisation of endometriosis lesions, which are more frequent on the left side in the pelvis and on the right side of the diaphragm since peritoneal fluid circulates [47] clockwise from the pouch of Douglas over the right gutter to the right diaphragm. The lower recurrence rate of endometriosis after surgery in women taking medical therapy might be explained by the lower menstrual volume or absence of menstruation. We can only speculate to what extent the severity of dysmenorrhoea might reflect the volume of retrograde menstruation and the acute inflammation, at least in some women [48].

It is unclear and may be considered speculative whether the mildly elevated CRP concentration, reflecting retrograde menstruation and mild acute inflammation, could be a cofactor in adhesion formation after surgery. This seems unlikely since surgery itself will cause a much more important acute inflammation of the peritoneal cavity by exposing the mesothelial cells to CO_2_ or to the oxidative stress of air and surgical manipulation. However, in mice, mild manipulation of the bowels in the upper abdomen enhances adhesion formation between opposing lesions in the lower abdomen [49].

Microbiota from the uterine cavity and upper genital tract may contribute to peritoneal inflammation caused by retrograde menstruation, as women with endometritis foci have more postoperative adhesions [50]. Similarly, an association with vaginal infections or vaginoses, as suggested for endometriosis, cannot be excluded [14]. Women with foci of endometritis have more postoperative adhesions without clinical symptoms of pelvic infection [50]. Indirect evidence thus suggests a relationship between the upper genital tract microbiome and the peritoneal inflammatory reaction [51].

The clinical consequences of mildly elevated CRP levels during menstruation are unclear and speculative. The clinician could consider the mild average increase in the average woman as clinically irrelevant. However, it might be wise to consider measuring CRP concentrations in women with abundant menstruation and dysmenorrhoea and, although speculative, to offer a treatment reducing menstrual flow to the individual woman with much more pronounced CRP increases. Similarly, it might be wise to avoid surgery during menstruation in women with very high CRP concentrations. Unfortunately, we have no data on whether anti-inflammatory drugs or other CRP-lowering agents [52] could be considered.

With a little imagination, retrograde menstruation and increased CRP concentrations could be discussed differently. In mammals, menstruation, which occurs only in humans, some primates, one mouse strain, and some bats, is the exception without clear benefits. Today, women have over 400 menstruations in a lifetime, which is much more than the estimated 30 to 40 menstruations, 100 years ago. Retrograde menstruation has, to the best of our knowledge, been demonstrated only in women. After occlusion of the cervix, baboons develop haematometra without retrograde menstruation [53]. We risk underestimating the importance of the abdominal cavity’s direct connection to the vagina and of sperm cells transporting microorganisms on their tails. Therefore, it has been discussed that menstruation and retrograde menstruation might have some reproductive advantages in embryo selection, but at a price. It could be discussed whether avoiding menstruation in all women who are not trying to conceive might be beneficial [12,54]. Abolishing menstruation might reduce the risk of endometriosis, considered a consequence of many retrograde menstruations because of delaying childbirth, together with microbiota. It could be argued that oral contraception should be given continuously, knowing that the 21 + 7 regimens with bleeding were chosen mainly to exclude an eventual pregnancy. In women without a pregnancy wish, tubal ligation could be reconsidered, primarily since it can be performed as a 5-min procedure under local anaesthesia [55]. Preventing menstruation and retrograde menstruation and preventing vaginal microbiota from reaching the abdominal cavity might turn out to reduce the risk of ovarian cancer since the incidence of ovarian cancer is decreased by 50% after tubal ligation [56,57] or long-term oral contraceptive use [58,59]. Half and a quarter dose of ovulation inhibition is likely sufficient in the majority of women, permitting individualisation of therapy [12].

That CRP plasma concentrations are logarithmically distributed, like most hormone concentrations [37,60,61], has no clinical implications, aside from preventing means minus 3 SDs from being negative.

## 5. Conclusions

The CRP concentrations increase during menstruation in most women. This increase is probably due to retrograde menstruation, which induces mesothelial cell retraction and acute inflammation. It seems unlikely that this CRP increase is caused by the inflammation associated with menstrual shedding, since it persists in the early follicular phase. Also, a contribution to the inflammatory reaction from the microbiota in the upper genital tract, the uterus, and eventually the vagina cannot be excluded. However, it should be stressed that, to date, this remains a hypothesis. Although minimal amounts of blood are sufficient for mesothelial cell retraction and acute inflammation in mice, it remains to be demonstrated that, in women, retrograde menstruation causes increased plasma CRP concentrations. It can only be speculated that CRP concentrations do not increase in women with occluded oviducts, and that the increase in CRP concentrations will be related to the volume of retrograde menstruation and thus probably to dysmenorrhoea and heavy menstrual bleeding. However, the hypothesis that an increase in menstrual CRP concentration reflects retrograde menstruation could be clinically important. First, mesothelial cell retraction likely facilitates endometrial cell implantation and could explain the preferential localisation of endometriosis lesions in the pelvis and on the right diaphragm. The clinician could consider individualisation of therapy in women with menorrhagia and dysmenorrhoea and severely increased menstrual CRP concentrations to reduce the implantation of viable endometrial cells or to prevent recurrences of endometriosis after surgery [9]. Second, it is speculative and remains to be demonstrated that the increased CRP concentrations might be a cofactor enhancing adhesion formation after surgery during menstruation. A third, rather philosophical aspect is menstruation, which can be considered as having some reproductive advantages but at a price. It could be argued that avoiding menstrual bleeding in women not planning to conceive could be beneficial for most [12,54].

## Data Availability

Available with the authors on simple request.

## Supplementary Materials

The following supporting information can be downloaded at: https://www.mdpi.com/article/10.3390/jcm15051711/s1. Figure S1: PRISMA 2020 for Flowchart.

## Abbreviations

The following abbreviations are used in this manuscript:

CRP	C-reactive protein
SD	Standard deviation
PMS	Premenstrual syndrome
EF	Early follicular
LF	Late follicular
L	Luteal
SQR	Semi Quartile Range
PCOS	Polycystic ovarian syndrome
