# Supplementary figures and images for: Increased C-Reactive Protein Concentrations During Menstruation May Be Important for the Pathophysiology of Endometriosis and Possibly for Adhesion Formation—A Systematic Review
Source: J Clin Med. 2026 Feb 24;15(5):1711. doi: 10.3390/jcm15051711 (PMC12985487; doi:10.3390/jcm15051711)

## Supplemental Material

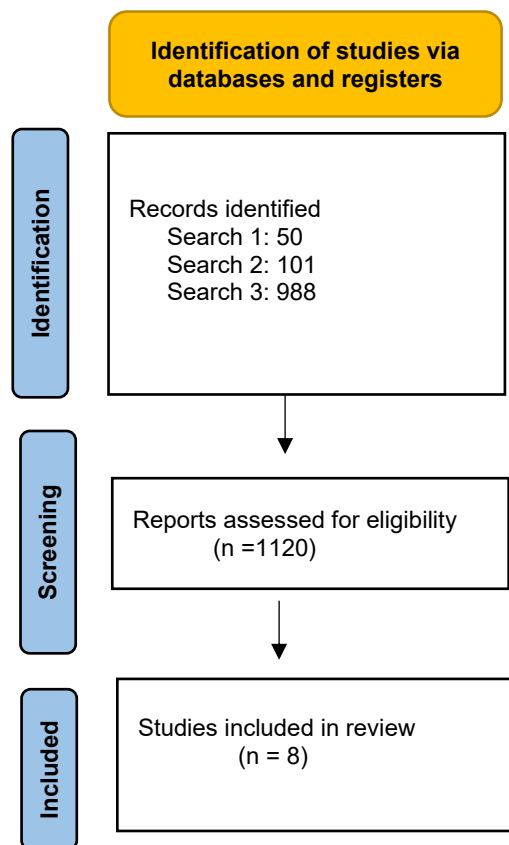

**Figure S1.** PRISMA 2020 for Flowchart

Supplement: Supplementary file 1 [file jcm-15-01711-s001.zip › jcm-4069337-supplementary.pdf]
